# Cost-effectiveness of physical activity intervention in children – results based on the Physical Activity and Nutrition in Children (PANIC) study

**DOI:** 10.1186/s12966-021-01181-0

**Published:** 2021-09-06

**Authors:** Virpi Kuvaja-Köllner, Niina Lintu, Virpi Lindi, Elisa Rissanen, Aino-Maija Eloranta, Sanna Kiiskinen, Janne Martikainen, Eila Kankaanpää, Hannu Valtonen, Timo A. Lakka

**Affiliations:** 1 Department of Health and Social Management, University of Eastern Finland, Kuopio, Finland; 2 Institute of Biomedicine, School of Medicine, University of Eastern Finland, Kuopio, Finland; 3 Library, University of Eastern Finland, Kuopio, Finland; 4 Institute of Public Health and Clinical Nutrition, School of Medicine, University of Eastern Finland, Kuopio, Finland; 5 School of Pharmacy, University of Eastern Finland, Kuopio, Finland; 6 Kuopio Research Institute of Exercise Medicine, Kuopio, Finland; 7 Department of Clinical Physiology and Nuclear Medicine, Kuopio University Hospital, Kuopio, Finland

**Keywords:** Physical activity, Children, Cost-effectiveness, Intervention, Multicomponent, Family, School, Net monetary benefit

## Abstract

**Background:**

We assessed the cost-effectiveness of a 2-year physical activity (PA) intervention combining family-based PA counselling and after-school exercise clubs in primary-school children compared to no intervention from an extended service payer’s perspective.

**Methods:**

The participants included 506 children (245 girls, 261 boys) allocated to an intervention group (306 children, 60 %) and a control group (200 children, 40 %). The children and their parents in the intervention group had six PA counselling visits, and the children also had the opportunity to participate in after-school exercise clubs. The control group received verbal and written advice on health-improving PA at baseline. A change in total PA over two years was used as the outcome measure. Intervention costs included those related to the family-based PA counselling, the after-school exercise clubs, and the parents’ taking time off to travel to and participate in the counselling. The cost-effectiveness analyses were performed using the intention-to-treat principle. The costs per increased PA hour (incremental cost-effectiveness ratio, ICER) were based on net monetary benefit (NMB) regression adjusted for baseline PA and background variables. The results are presented with NMB and cost-effectiveness acceptability curves.

**Results:**

Over two years, total PA increased on average by 108 h in the intervention group (95 % confidence interval [CI] from 95 to 121, *p* < 0.001) and decreased by 65.5 h (95 % CI from 81.7 to 48.3, *p* < 0.001) in the control group, the difference being 173.7 h. the incremental effectiveness was 87 (173/2) hours. For two years, the intervention costs were €619 without parents’ time use costs and €860 with these costs. The costs per increased PA hour were €6.21 without and €8.62 with these costs. The willingness to pay required for 95 % probability of cost-effectiveness was €14 and €19 with these costs. The sensitivity analyses revealed that the ICER without assuming this linear change in PA were €3.10 and €4.31.

**Conclusions:**

The PA intervention would be cost-effective compared to no intervention among children if the service payer’s willingness-to-pay for a 1-hour increase in PA is €8.62 with parents’ time costs.

**Trial registration:**

ClinicalTrials.gov: NCT01803776. Registered 4 March 2013 - Retrospectively registered, https://clinicaltrials.gov/ct2/results?cond=&term=01803776&cntry=&state=&city=&dist=.

**Supplementary Information:**

The online version contains supplementary material available at 10.1186/s12966-021-01181-0.

## Introduction

Low levels of physical activity (PA) have been associated with increased risk of various chronic diseases and therefore with increased health care costs [1, 2]. PA habits are often formed early in life [3–6], and lifestyle-related chronic diseases, such as type 2 diabetes, start to develop already during the fetal period [7]. According to a recent review on PA interventions carried out in childhood, parents play a key role in promoting their children’s PA [8]. Multicomponent interventions, such as school-based interventions in combination with involvement of families or communities, have also been found to be effective in increasing PA among adolescents [8–10].

Healthcare decision makers increasingly require scientific evidence to support their decisions on the allocation of available resources [11]. Economic evaluation comparing the costs and effectiveness of different interventions helps in identifying the best option for the efficient use of healthcare resources [12]. The key question in economic evaluation is whether the intervention represents “value for money” [13]. Although there have been a large number of studies to investigate the effectiveness of PA interventions in increasing PA and thereby improving health in children, only a few of them have included economic evaluation [14–19]. Moreover, many studies reporting the cost-effectiveness of lifestyle interventions have been carried out only among overweight children [18, 19]. Another option to evaluate the economic consequences of interventions would be to use a modelling study based on the results of an effectiveness study [20–22].

There is some evidence for the cost-effectiveness of school-based PA interventions [23], particularly when no extra-staff was required to carry out the interventions [17]. However, little is known about the cost-effectiveness of family-based PA interventions. We therefore assessed the cost-effectiveness of a 2-year family-based PA intervention from the perspective of a municipality as a service payer and additionally extended the analyses by including parents’ time use costs in a general population of primary-school children.

## Methods

### Study design and study population

The Physical Activity and Nutrition in Children (PANIC) study is a controlled trial on the effects of a combined PA and dietary intervention on cardiometabolic risk factors and other health outcomes in a population sample of children from the city of Kuopio, Finland [24, 25]. The Research Ethics Committee of the Hospital District of Northern Savo approved the study protocol in 2006 (Statement 69/2006). The parents or caregivers of the children gave their written informed consent, and the children provided their assent to participation. The PANIC study has been carried out in accordance with the principles of the Declaration of Helsinki as revised in 2008.

We invited 736 children aged 6–9 years who started the first grade in 16 primary schools of the city of Kuopio in 2007–2009 to participate in the study (Additional file 1). Altogether, 512 (70 %) children (248 girls, 264 boys) accepted the invitation and participated in the baseline examinations between October 2007 and December 2009. The participants did not differ in sex, age, height – standard deviation score (SDS) or body mass index (BMI) - SDS from all children who started the first grade in the city of Kuopio in 2007–2009. We excluded six children from the study at baseline either because of physical disabilities that could hamper participation in the intervention or who had no time or motivation to attend the study. The final study sample thus included 506 children at baseline.

We allocated the children from nine schools to a combined PA and dietary intervention group (306 children, 60 %) and the children from seven schools to a control group (200 children, 40 %) to avoid contamination in the control group by after-school exercise clubs organised in the nine schools or any local or national health promotion programmes that could have been initiated in the study region during the follow-up period. We also proportionally matched the intervention and control group according to the location of the schools (urban vs. rural) to minimise sociodemographic differences between the groups. We included more children in the intervention group than in the control group because of a larger number of dropouts expected in the intervention group and to retain a sufficient statistical power for comparison between the groups. A total of 261 children (85 % of those invited) from the intervention group and 179 (90 %) children from the control group participated in the 2-year follow-up examinations between November 2009 and January 2012. The median (interquartile range) of follow-up time was 2.1 (2.1–2.2) years in the intervention and control group. Data on PA used in the analyses were available for 503 children (244 girls, 259 boys) at baseline and for 431 children (210 girls, 221 boys) at 2-year follow-up. (Flow chart Additional file 1)

### Physical activity intervention

The 2-year PA intervention included six family-based and tailored PA counselling visits organised during office hours for each family [26]. The children and their parents received individual advice from an exercise medicine specialist on how to increase PA and decrease sedentary time of the children in everyday conditions. Each visit had a specific topic of discussion in accordance with the goals of the intervention and included practical tasks on these topics for the children. The families also received fact sheets on PA and sedentary time as well as verbal and written information on opportunities for exercising in the city of Kuopio. Minor gifts, such as exercise equipment and admission to indoor sports facilities, were given for all families to support PA of the children. The timing and topics of and the time of exercise medicine specialists used for the family-based and tailored PA counselling visits are presented in Table 1. Of the 306 children in the intervention group who attended the baseline examination, 266 (87 %) participated in all six visits, 281 (92 %) in at least five visits, and 295 (96 %) in at least four visits.

**Table 1:** Timing and topics of the family-based and tailored physical activity counselling visits

Timing of the visits	Main topics of the visits	Time used for the visits
0.5 months after baseline	Introducing the families to the content of the intervention and an overview of a physically active lifestyle	15 min
1.5 months after baseline	Supporting spontaneous physical activity in children	70 min
3 months after baseline	Supporting the aim of achieving recommended amount of physical activity, sedentary time, and sleep	30 min
6 months after baseline	Increasing physical activity in everyday life and with the family	35 min
12 months after baseline	Supporting the development of motor skills	40 min
18 months after baseline	Health benefits of a physically active lifestyle	30 min
Total time used		220 min

The children in the intervention group, particularly those who did not attend organised sports or exercise, were also encouraged to participate in after-school exercise clubs organised at the nine schools by trained exercise instructors of the PANIC study. There were a total of 24 after-school exercise clubs that lasted 60 min and took place on average once a week. Altogether, 254 (87 %) of the 306 children in the intervention group participated in at least one of the after-school exercise club sessions, and 124 (41 %) of these children attended the exercise clubs at least once a month. The children participated in on average 23 (95 % confidence interval 20–26) of all 76 exercise club sessions.

In the control group, the children and their parents received general verbal and written advice on health-improving PA at baseline but no further PA counselling. The children in the control group normally participated in the compulsory 1.5 h of physical education per week, but they were not allowed to attend the after-school exercise clubs to avoid a non-intentional intervention in the control group.

### Assessment of physical activity

Total time used for PA during a usual week was assessed using the PANIC Physical Activity Questionnaire, filled out by the parents or caregivers at baseline and at 2-year follow-up [27]. This questionnaire has been validated in a subsample of children from the PANIC Study by the Actiheart® monitor [28]. The types of PA in the questionnaire included unsupervised PA, organised sports, organised exercise other than sports, physically active school transportation, and PA during recess. Total weekly PA was calculated by summing the time spent for different types of PA. The compulsory 1.5 h of physical education per week for all children aged 7–15 years in Finnish schools was included in total PA.

### Assessment of socioeconomic background

Socioeconomic status, including parental education and household income, was reported by the parents or caregivers at baseline. The degree of the more educated parent was used as parental education in the analysis [29]. Household income was divided into three categories (≤ 30,000 €/year, 30,001–60,000 €/year, and ≥ 60,001 €/year).

### Resources used and related costs

The costs of the PA intervention included those related to the family-based PA counselling visits, the after-school exercise club sessions, and the parents’ or the caregivers’ taking time off for traveling to and participating in the counselling, but not those related to planning the PA intervention. In the cost-effectiveness analyses, the costs of the PA intervention were assumed to be similar for all participants in the intervention group, and those for the control group were assumed to be zero.

The costs of the family-based PA counselling visits included the salary of the exercise medicine specialist; the printed material for the parents or the caregivers; minor gifts, such as exercise equipment and admission to indoor sports facilities, to support PA of the children; and healthy snacks served to the children during the PA counselling visits. The salary costs for the exercise medicine specialists are based on the time used for PA counselling and the average earnings for a person with a Master’s degree in health sciences working in the municipal sector in Finland in 2011 of 3405 €/month [30] added by all social security costs of 40 % [31].

Since the family-based PA counselling visits were organised during office hours, we assumed that one parent or caregiver needed to take time off to pick up the child from school and attend the visits. The estimated working time lost was valued according to the opportunity cost approach [12, 32]. We estimated that each parent spent altogether 8.67 h for the PA counselling, calculated as 2.67 h (220 min) for the six counselling visits + six hours for transportation to the six counselling visits (one hour for each visit). The costs of time used for the parents were valued using the average Finnish employee’s earnings of 19.84 €/hour added by all social security costs of 40 % in Finland in 2014 [31, 33].

The after-school exercise club sessions lasted 60 min, and the trained exercise instructors used another 30 min for planning the sessions and transferring equipment. The salary costs of the exercise instructors were valued using the earnings of these employee in the city of Kuopio of 2246 €/month added by all social security costs of 40 % in Finland in 2014 [31]. A school year in Finland includes 38 school weeks, so there were approximately 76 after-school exercise club sessions over two years in the 24 exercise clubs. For the analyses, the costs of the 76 exercise club sessions were multiplied by the number of the after-school exercise clubs of 24, and the value was divided by the number of children in the intervention group of 306. The city of Kuopio provided the information used for the rental costs of school sports halls. There was large variation in the rental costs, and therefore their average was used in the analyses.

### Statistical methods

All statistical analyses were performed using Stata 15 [34]. Differences in baseline characteristics, including gender, age, education, and income, between the intervention group and the control group as well as between children who completed the intervention and those who did not were analysed using the t-test for independent samples for normally distributed continuous variables and the Chi-squared test for categorical variables. The cost-effectiveness analyses were performed following the intention-to-treat principle [35]. Chained equation was used to impute missing values for PA [36, 37]. In these data, we assumed that the values were missing at random. Predictive mean matching with the five closest cases was used in the imputation model [38]. Gender, age, school, parental education, and household income at baseline were used as predictors to impute missing PA values at baseline and at 2-year follow-up. We had complete data on PA and background variables at baseline and at follow-up for 83 % of the children and thus partly incomplete information for 17 % of the children. The number of imputed datasets has been recommended to be similar to the percentage of study participants with partly incomplete information [39]. Therefore, 17 new datasets were imputed. The results of all imputed datasets were combined with other data using the Rubin’s rules [38].

### Economic evaluation

For the economic evaluation, the weekly hours of PA at baseline and at 2-year follow-up were spread over the 2-year period to be comparable with the costs of the PA intervention over two years. Because it was not obvious when the change in PA took place during the two years, we assumed that it occurred linearly and used the trapezoidal rule [40, 41] and divided the area under the curve (area between baseline and 2-years measurement) by two, as shown in the following calculation formula. The change in PA over two years was calculated as (104 weeks * PA hours per week at 2-year follow-up)/2 – (104 weeks * PA hours per week at baseline)/2.

The economic evaluation was performed using the net monetary benefit analysis with two main steps. Firstly, we calculated the net monetary benefit for each child in the dataset using the formula the net monetary benefit = λ * E_i_-C_i_, where λ represents the threshold for willingness to pay for the specified outcome, E_i_ is the observed effect for the subject, and C_i_ is the costs for the subject i [42]. Secondly, we performed regression analysis using each child’s net monetary benefit as the dependent variable. The regression coefficient δti for the treatment dummy variable (1 = intervention, 0 = control) provides the estimate of the incremental net monetary benefit. When δti is higher than 0, the incremental net monetary benefit is positive, and the intervention is cost-effective compared with control [42–44]. Other variables in the regression model were the intercept α and the stochastic error ε_i_ [45, 46]. PA, gender, age, parental education, and household income at baseline were added as independent variables and school at baseline was added as a cluster variable into the net benefit regression model [45, 46].

Net monetary benefit =〖α + δt〗_i + baseline PA + baseline age + gender + baseline parental education + baseline household income + ε_i_ {school as cluster variable}.

Cost-effectiveness acceptability curves were used to characterize uncertainty in the net monetary benefit according to willingness to pay for 1-hour increase in PA [12, 42, 46, 47]. The results are also presented as incremental cost-effectiveness ratios, denoting the extra costs per an extra unit of effect, which can be found from the point where the incremental net monetary benefit turns positive [45].

### Sensitivity analysis

To assess the robustness of the results, five sensitivity analyses were conducted. First, we tested whether the results would change if the costs were 20 % higher. Second, as the most pessimistic option, we combined 20 % lower effectiveness with 20 % higher costs of the intervention. Third, we discounted the costs and the effectiveness by 3 % [48]. Fourth, we included only children with complete data in the analyses. Fifth, as the most optimistic option, we assumed an immediate change in PA after the start of the follow-up. These sensitivity analyses were performed using the net monetary benefit regression, and the results are presented as threshold values with a probability of 50 % of cost-effectiveness. All sensitivity analyses were performed by considering parents’ time used and without it.

## Results

### Characteristics of children

The self-reported household income at baseline was statistically significantly higher in the intervention group than in the control group (Table 2). There were no differences in total PA or other baseline variables between the groups. There were more children in the lowest third of parental education and household income among non-completers than among completers in both groups (p<0.001).

**Table 2:** Background characteristics of children at baseline

	Intervention group (*n* = 306)	Control group (*n* = 200)	*P*-value
Boys, n (%)	162 (53)	99 (49)	0.462
Age, years (SD)	7.6 (0.4)	7.6 (0.4)	0.399
Parental education, n (%)			0.564
Vocational school or less	57 (19)	43 (21)	
Polytechnic	139 (46)	85 (43)	
University	107 (35)	72 (36)	
Household income, n (%)			0.019
≤ 30,000 €	51 (17)	55 (28)	
30,001–60,000 €	132 (43)	76 (39)	
≥ 60,001 €	117 (38)	65 (33)	

The values are means (standard deviations) from the T-test for independent samples for age and numbers (percentages) from the Chi-squared test for parental education and household income.

### Cost-effectiveness

PA increased on average by 63 min per week (standard deviation [SD] 281, 95 % confidence interval [CI] 55 to 70, *p* < 0.001) in the intervention group and decreased by 38 min per week (SD 294, 95 % CI 47 to 28, *p* < 0.001) in the control group over two years. Families in the highest third of household income were more likely to participate in all six PA counselling visits than families in the lowest third (*p* = 0.015).

PA increased on average by 108.2 h per child over two years in the intervention group and decreased average by 65.5 h per child over two years in the control group by using imputed and adjusted PA values in the economic evaluation (Table 3). Thus, the incremental effectiveness of the PA intervention was 173.7 h (108.2 h - (-65.5) hours) over two years (*p* < 0.001), and the linear effectiveness of the PA (173.7/2) intervention was 86.9 h over two years (*p* < 0.001).

**Table 3:** Imputed and adjusted physical activity values in the intervention group and in the control group

	Average of physical activity hours per child over two years	Values used in economic analysis^a^ (95 confidence intervals)
Intervention group		
Baseline	1369 h ^b^	
2-year follow-up	1477 h ^c^	
Change over 2 years	108.2 h	54.0 h (47.6 to 60.5 h)
Control group		
Baseline	1306 h ^d^	
2-year follow-up	1241 h ^e^	
Change over 2 years	-65.5	− 32.8 h (-40.9 to − 24.2 h)
Incremental effectiveness	173.7 h ^f^	86.8 h (76.1 to 97.0 h)

^a^Linear change in PA assumed, the trapezoidal rule applied, and change in PA divided by 2

^b^13.16 h/w * 104 weeks =1368.64 hours,

^c^14.2 h/w * 104 weeks=1476.80 hours,

^d^12.56 h/w * 104 weeks =1306.24 hours,

^e^11.93 h/w * 104 weeks=1240.72 hours,

^f^108.16 – (-65.52) = 173.68 hours

The costs of the PA intervention, reflecting the resources used, were 619 € per child over two years without parents’ time use costs (Table 4) and 860 € per child with parents’ time use costs. Most of the costs were related to organizing after-school exercise clubs, such as personnel costs and rentals. Moreover, costs related to parents’ time used played a major role. Other costs, such as those related to family-based PA counselling, were minor.

**Table 4:** Resources used for the physical activity intervention and associated costs over two years

	Resources used	Unit cost	Cost per exercise club	Cost per child
Family-based physical activity counselling (6 visits)				
Personnel costs of family-based counselling ^a^	3.67 h	€29.56		€108.50
Printing costs of fact sheets given for the families	20 pcs	€0.08		€1.52
Minor gifts for the families				€20.60
Healthy snacks served to the children				€2.70
Parents’ time use costs ^b^	8.67 h	€27.78		€240.88
After-school exercise clubs				
Personnel costs of after-school exercise clubs ^c^	76.00 h ^d^	€58.49	€4445.50 ^e^	€348.67 ^f^
Costs of sports halls	76.00 h ^d^	€23.05	€1751.80 ^g^	€137.40 ^h^
All costs per child without parents’ time use				€619.00
All costs per child with parents’ time use				€860.27

^a^) Personnel costs: exercise medicine specialist with average hourly wage 21.12€ + 40% overhead= €29.56/h

^b^) Parents’ time use costs. Average hourly wage in year 2014 = €19.84 + 40% overhead = €27.78/h

^c^) 2 exercise instructors * 1.5 hours* (€13.93 + 40% overhead) = 2 * 1.5 hours * €19.50 = €58.49 per exercise club session

^d^) There were 38 exercise clubs per year and thus 76 exercise clubs during the 2-year intervention.

^e^) Personnel costs for after-school exercise clubs (€58.49 * 76 times) = €4445.50 per group

^f^) (24 groups * €4445,50)/306 children= €106 692/ 306 children = €348.67 per child

^g^) Costs of sports hall (€23.05 * 76 times) = €1751.80 per group

^h^) (24 groups * €1751.80)/306 children = €42043.20/ 306 children= €137.40 per child

**Fig. 1:**
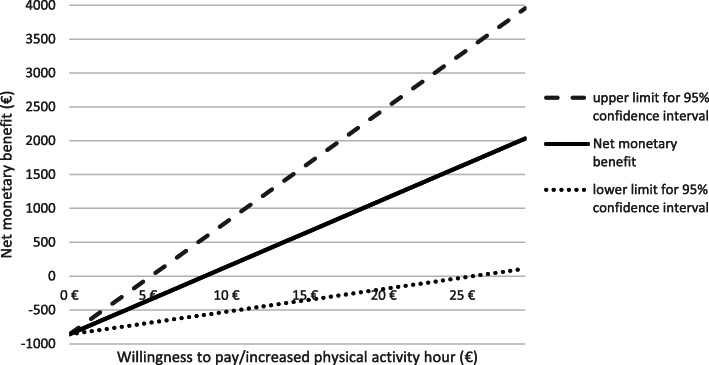
Net monetary benefit with parents’ time use costs in relation to willingness to pay for 1-hour increase in PA)

**Fig. 2:**
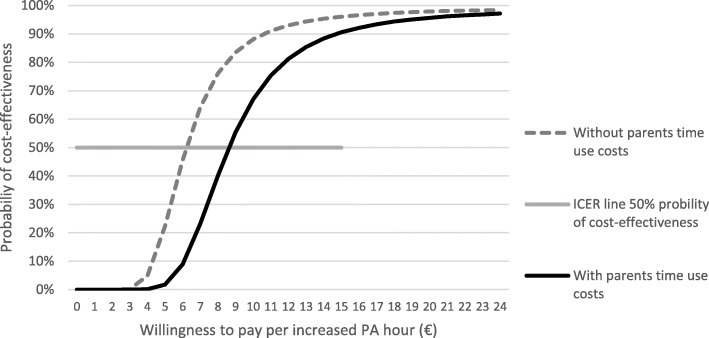
Cost-effectiveness acceptability curves for 1-hour increase of PA showing the probability that the intervention is cost-effective compared to the control

The PA intervention resulted in an incremental benefit of 87 h in PA and incremental program costs of €619 without parents’ time use costs and €860 with these costs over two years. The net monetary benefit of the PA intervention, indicated by the point where the curve intersects the x-axis in Fig. 1, is positive when willingness to pay for a 1-hour increase in PA is at least €8.62 with parents’ time use costs (Fig. 1). Without these costs, the net monetary benefit of the PA intervention turns positive when the willingness to pay for a 1-hour increase in PA is at least €6.21 (Additional file 2). The willingness to pay required for 95 % probability of cost-effectiveness was €14 without parents’ time use costs and €19 with these costs (Fig. 2).

The cost per a 1-hour increase in PA varied between €4.41 and €12.42 in the sensitivity analyses (Additional file 3). The effectiveness of the PA intervention had the largest impact on incremental cost-effectiveness ratios. If we assumed that PA increased immediately after the initiation of the PA intervention, the net monetary benefit turns positive at €4.31.The results remained similar when using the intention-to-treat principle and when including only children with complete data, as indicated by the overlapping lines (Additional file 3). The incremental cost-effectiveness ratios were €2–3 lower without costs related to parents’ time use.

## Discussion

We assessed the cost-effectiveness of the 2-year PA intervention compared to no intervention in a general population of primary-school children aged 6–9 years. The PA intervention included six family-based and tailored PA counselling visits for all families in the intervention group and after-school exercise clubs, particularly for children in the intervention group who did not attend organised sports or exercise. The cost-effectiveness analysis was performed from an extended service payer’s perspective, with and without parents’ time costs, by using imputed and adjusted PA values. PA was estimated to increase average by 54 h per child in the intervention group and to decrease average by 33 h per child in the control group over two years, the difference in PA change being 87 h over two years between the groups. The costs of the PA intervention over two years were €619 per child without parents’ time costs and €860 per child with these costs. The PA intervention is cost-effective compared to no intervention among children if the service payer’s willingness-to-pay for a 1-hour increase in PA was €6.21 without parents’ time costs and €8.62 with these costs.

The cost of increased PA hour was estimated by the incremental cost-effectiveness ratio (ICER) and was based on the net monetary benefit regression analyses assuming a linear change in PA. Our results mean that if the service payers are satisfied with a 50 % probability that the PA intervention is cost-effective compared with no intervention, the willingness to pay needs to be €6.21 per increased PA hour without parents’ time costs and €8.62 with them. If they want to have at least 95 % probability that the PA intervention is cost-effective, their willingness to pay needs to be €14 without parents’ time costs and €19 with these costs. There is no generally accepted willingness-to-pay value for an hour increase in PA for children, so it makes sense to compare the costs of our PA intervention with those of other activities offered by the service payers, such as municipalities and parents. Swimming halls in Finland are mainly operated and subsidised by municipalities, and the cost of using swimming halls is estimated to be about €6 per visit [49, 50]. The swimming hall ticket costs €4 for a child and €6.50 for an adult, resulting in a total cost of €10.50 per visit. The costs of health-promotion services in the private sector tend to be more expensive than those provided by municipalities. The sensitivity analyses revealed that the cost of an increased PA hour without assuming a linear change (halving PA hours) in PA would be €3.10 without parents’ time costs and €4.31 with these costs. In other words, halving intervention effectiveness increased the cost per PA hour gained. Moreover, the rental costs of sport halls vary widely in Finland, and higher costs would markedly increase the costs of the increased PA hour.

There is some evidence supporting the cost-effectiveness of PA interventions combining the involvement of school and parents and targeting children [14, 17, 21, 22]. For example, a new playscape installation in the metropolitan parks of Melbourne, Australia, resulted in increased PA and was observed to be cost-effective [14]. Among children, there was a net increase of 68 884 metabolic equivalent (MET) hours over 14 months in the intervention group compared to the control group, together with other age groups, this intervention yield costs of 0.58 AUD$ per MET-hour gained per visitor. Another Australian school-based PA intervention targeting adolescents was also found to be cost-effective with an ICER as high as 56 AUD$ per an additional minute of moderate-to-vigorous PA gained per day [15]. The results of our study are not directly comparable to these findings because we had a family-based PA intervention for all families combined with after-school exercise clubs, particularly for children in the intervention group who did not attend organised sports or exercise, in a general population of Finnish children, and we used total PA hours as the outcome measure in our cost-effectiveness analyses. We did not have the information about the intensity of PA, so the use of metabolic equivalent [51] units and their thresholds for cost-effectiveness [17] was not possible. One option would be to compare the cost-effectiveness ratios of different PA interventions by using the Relative Value Index [52]. However, there is no previous evidence on the cost-effectiveness of PA interventions in general populations of children aged 6–9 years like in the PANIC study.

Because the focus in this paper was the cost-effectiveness, the PA was assessed as whole. The art and type of PA have been explored in other PANIC articles [25]. Total PA at baseline was, on average, 1.9 h per day in our sample of children aged 6–9 years. When the PANIC study started in 2007, children of this age in Finland were recommended to have at least two hours of PA per day according to the national PA [53]. This recommendation was met by 40 % of the children in the intervention group and by 39 % of the children in the control group at baseline. At the 2-year follow-up, these proportions were 67 and 46 % in favour of the intervention group. According to a recent review, children’s PA decreases during the first school years [54], and the number of drop-outs in intervention studies among children with a lower socio-economic status is quite common [55]. Consistent with this finding, there were more drop-outs among families with lower education and household income in both groups. We do not have data on families that did not participate in our study, so we do not know whether socio-economic status affected the decision to attend. Parents usually make the decision whether to participate and continue in lifestyle interventions, particularly those including family-based lifestyle counselling. It may have been more difficult for parents with a lower education and household income to organise participation in the PA counselling visits because of their type of work, for example in a factory or a shop, than for parents with a higher socioeconomic status. Consistent with this assumption, children from families with lowest household income and education group were less likely to attend the PA counselling visits than those from higher-income and education families. The another explanation for these differences could be that individuals with a higher socioeconomic status (SES) are more health conscious and thus more likely to participate in health-related studies, such as the PANIC study, and also continue until the end of such studies than those with a lower SES [56]. We assumed that the parents needed to be absent from work due to the PA counselling visits, so their time costs were based on lost production. This assumption may cause overestimation of time costs because some parents may have shift work or may be unemployed, which could lower their costs of time. On the other hand, it may also result in underestimation of time costs if the parents were supposed to be asleep instead of working on a night shift or attending a job interview.

The costs of the family-based PA counselling visits were €133 (15 % of all costs), the parents’ time use costs were €241 (28 %), and the costs of the after-school exercise clubs were €486 (57 %). Although the after-school exercise clubs represented only about 20 % of the increased PA in the intervention group, they were the most expensive part of the PA intervention. This means that the family-based PA counselling visits rather than the after-school exercise clubs were cost-effective in our PA intervention. On the other hand, after-school exercise clubs are widely organised in Finland. The school halls in Finland are often available for after-school exercise clubs, especially in the early afternoon hours. In general, cooperation with schools should be part of the public health promotion strategy. In addition, most primary-school children aged 6–9 years need after-school care and many municipalities also organise afternoon care that is partly subsidized by the state. In most municipalities, parents are responsible for the afternoon care expenses, the monthly fee being €120–160 [57] for 4–5 h per day. Our findings suggest that the family-based PA counselling visits contributed more to the increased PA but were less expensive to organise than the after-school exercise clubs. Although it is more challenging to implement than the after-school exercise clubs, the family-based PA counselling, possibly combined with dietary counselling, could be tested and implemented in the school health care of Finland. Before starting such health promotion activities, school nurses should be trained in lifestyle counselling. Nevertheless, extensive co-operation and planning among scientists and public health promoters is needed to implement the lifestyle counselling as part of the established activities of municipalities.

The strengths of our study include the population-based sample of children studied and carrying out the PA intervention in a real-life setting. The careful economic evaluation of the data with a small number of drop-outs increased the reliability of the results. Moreover, the results using imputed and adjusted data and data on children who completed the 2-year intervention study were similar. The resources used for the PA intervention were well documented. We also used salaries and overheads based on national statistics that improved the reliability, generalisability, and transferability of the results. Another strength of our study is that the net monetary benefit regression approach was used. Especially with no official threshold for society’s or service provider’s willingness to pay, such as for a 1-hour increase in PA, this way of presenting the results is useful for decision makers and service payers. However, it would be easier to interpret the results if there was an agreement on the maximum willingness to pay for a 1-hour increase in PA.

This study also has some limitations. Firstly, PA was assessed only twice, at baseline and at the 2-year follow-up, whereas the costs of the PA intervention accumulated throughout the two years. For the economic evaluation purpose, the weekly PA hours at baseline and at the 2-year follow-up were spread over the 2-year period to be comparable to the costs of the PA intervention over two years. This required an estimate of the change in PA when it occurred. We wanted to avoid overly optimistic conclusions and thus halved the change in PA by applying the trapezoidal rule and spread the weekly PA hours for two years. However, this analysis approach had a major impact on the results by decreasing the effectiveness of the PA intervention and raised the incremental cost-effectiveness ratio per a 1-hour increase in PA. It would be important for researchers to discuss how to make the effectiveness and costs of the interventions comparable in economic analyses if there are only two measurements of PA, but the costs are spread unequally over the study period. Finally, we assessed PA using a questionnaire filled out by the parents in our general population of children instead of objective measures, such as accelerometers or combined heart rate and body movement monitors. The reason for choosing a questionnaire to assess PA in the present analyses is that we wanted to assess changes in the time spent in PA behaviour rather than changes in energy expenditure or PA intensity, for which objective measures would have been a better choice.

## Conclusions

Our study showed that the 2-year PA intervention combining family-based and tailored PA counselling and after-school exercise clubs was cost-effective in a general population of primary-school children. These findings provide further evidence that multicomponent PA interventions may be cost-effective in increasing PA among children. Our results suggest that the family-based PA counselling visits was a more cost-effective modality of the PA intervention than the after-school exercise clubs. It would therefore be important to test the effectiveness and cost-effectiveness of family-based PA counselling among children in health care. Our study also provides further evidence that it is more challenging to engage children from a lower socioeconomic background in long-term lifestyle intervention studies. The findings of our study are potentially important for the decision makers and service payers of health care when planning and implementing activities aimed at health promotion among children.

## Supplementary Information

**Additional file 1:**

**Additional file 2:**

**Additional file 3:**

## Data Availability

Data availability Information about the PANIC study and the data used in the present paper are available at www.panicstudy.fi/en/etusivu. The data are not publicly available due to research ethical reasons and because the owner of the data is the University of Eastern Finland and not the research group. However, the corresponding author can provide further information on the PANIC study and the PANIC data on a reasonable request.
