# Supplementary material for: Cost-effectiveness of physical activity intervention in children – results based on the Physical Activity and Nutrition in Children (PANIC) study
Source: Int J Behav Nutr Phys Act. 2021 Sep 6;18:116. doi: 10.1186/s12966-021-01181-0 (PMC8419957; doi:10.1186/s12966-021-01181-0)
Supplement: Supplementary file 1 — Additional file 1: [file 12966_2021_1181_MOESM1_ESM.pdf]

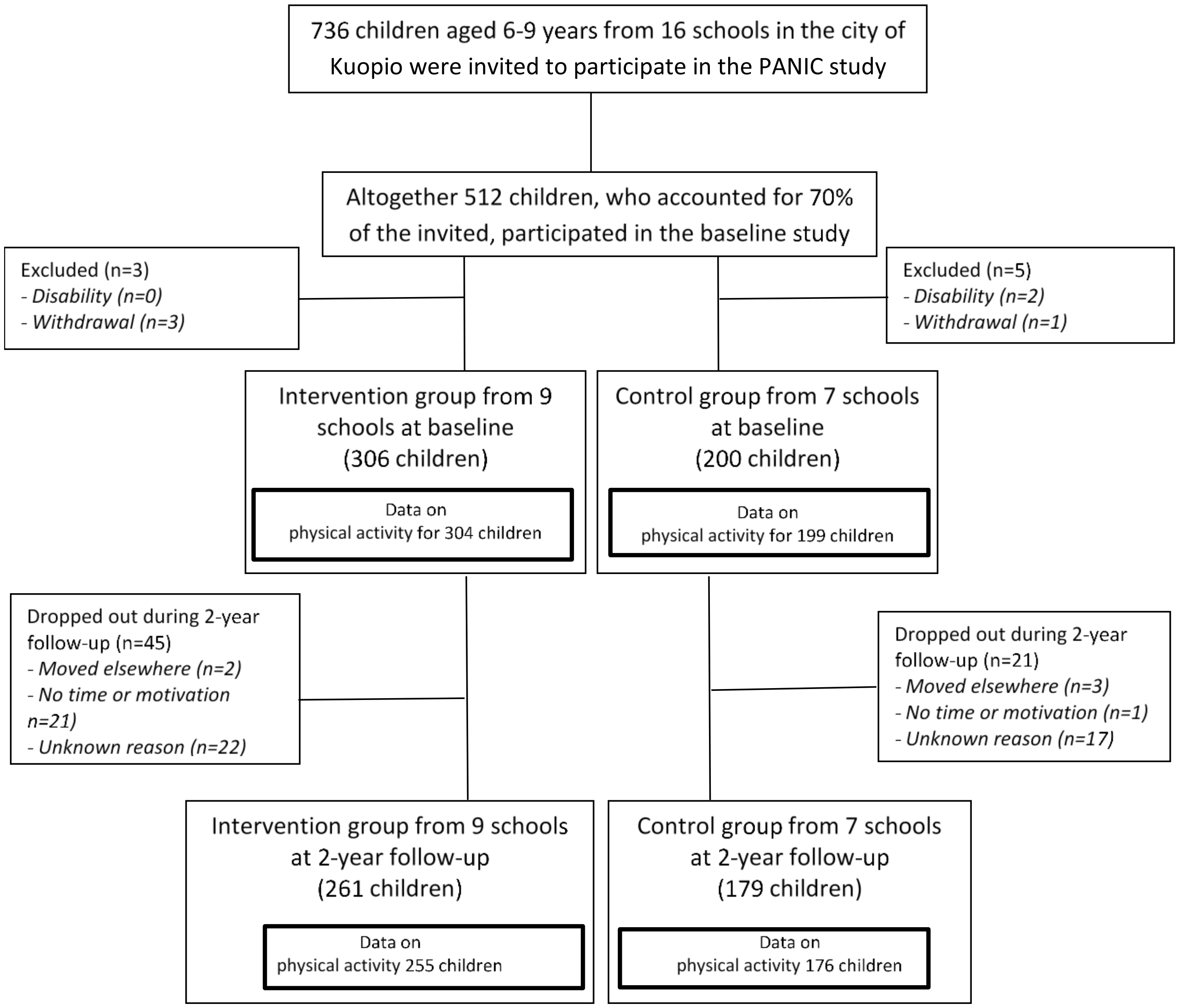

Fig1. Flow chart of the Physical Activity and Nutrition in Children (PANIC) Study in Finnish children.
