# Supplementary material for: Cost-effectiveness of physical activity intervention in children – results based on the Physical Activity and Nutrition in Children (PANIC) study
Source: Int J Behav Nutr Phys Act. 2021 Sep 6;18:116. doi: 10.1186/s12966-021-01181-0 (PMC8419957; doi:10.1186/s12966-021-01181-0)
Supplement: Supplementary file 2 — Additional file 2: [file 12966_2021_1181_MOESM2_ESM.pdf]

Additional File 2.

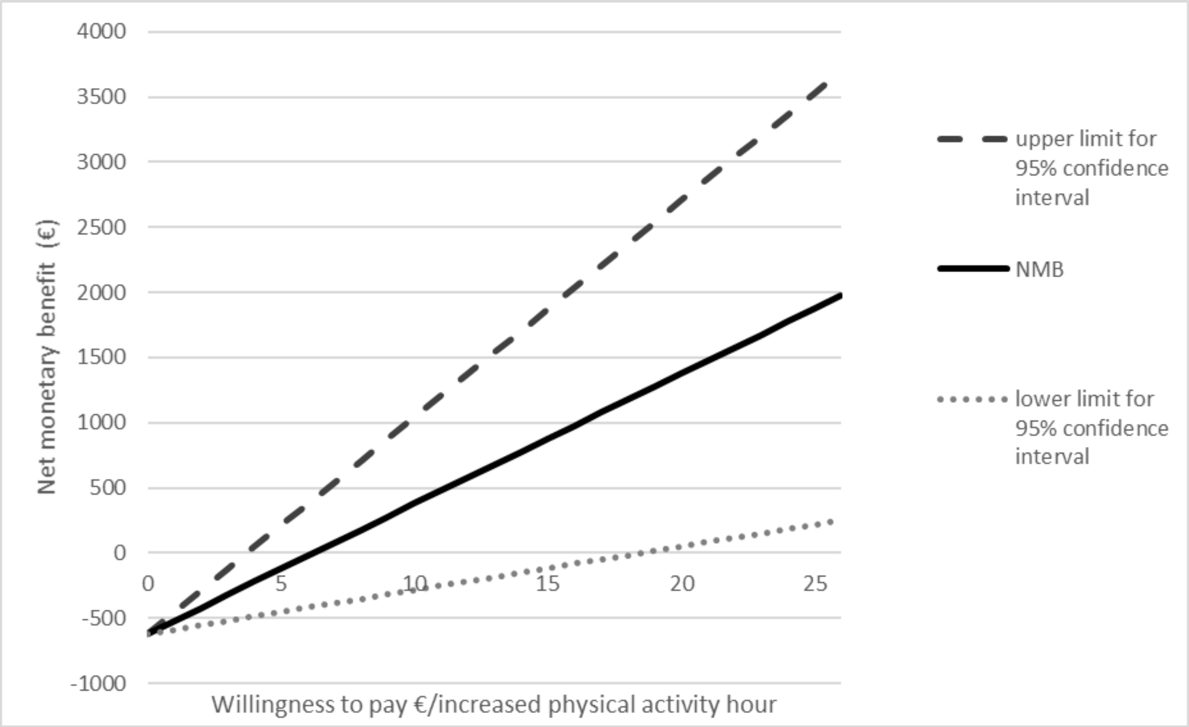

Fig. 1. Net monetary benefit in relation to willingness to pay for 1-hour increase in PA.
