# Supplementary material for: Cost-effectiveness of physical activity intervention in children – results based on the Physical Activity and Nutrition in Children (PANIC) study
Source: Int J Behav Nutr Phys Act. 2021 Sep 6;18:116. doi: 10.1186/s12966-021-01181-0 (PMC8419957; doi:10.1186/s12966-021-01181-0)
Supplement: Supplementary file 3 — Additional file 3: [file 12966_2021_1181_MOESM3_ESM.pdf]

Additional File 3.

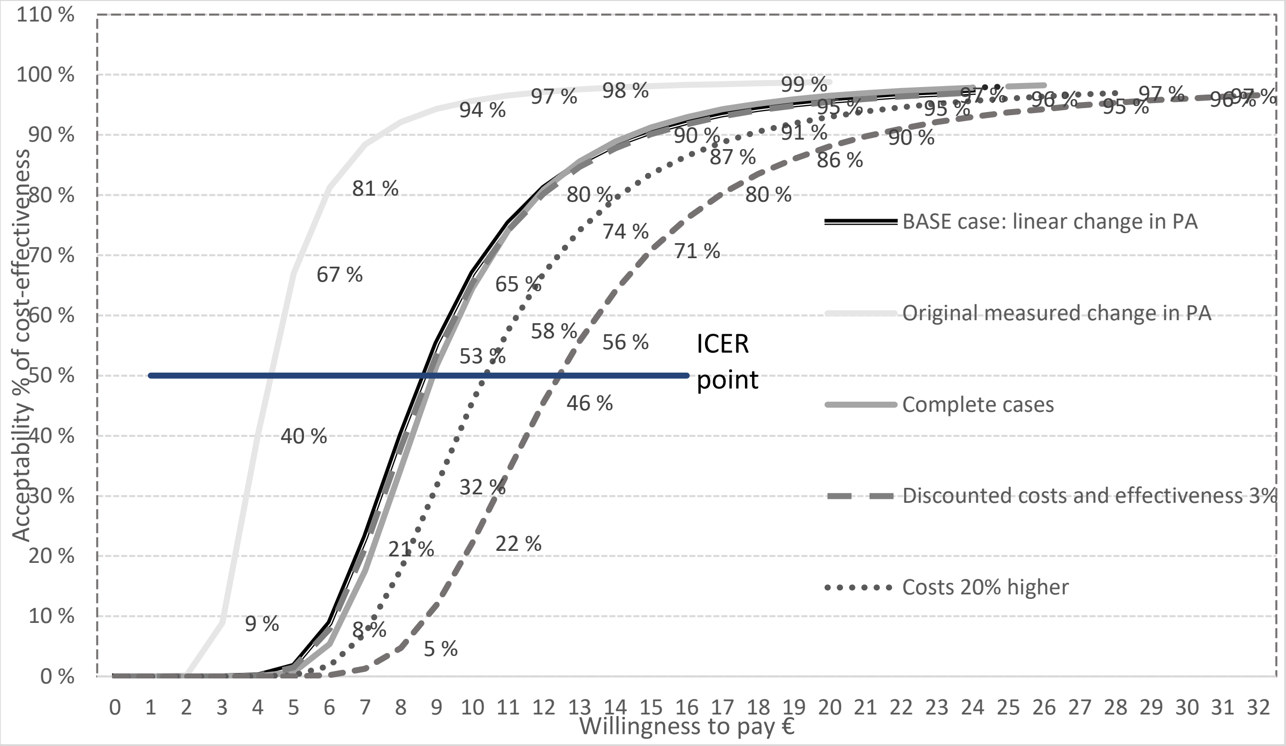

Figure 1. Cost-effectiveness acceptability curves for 1- h increase of PA showing the probability that the intervention is cost-effective under base case and various sensitivity analysis with assumptions of willingness to pay
